# Comparative study of the effects of diosmin and diosmetin on fat accumulation, dyslipidemia, and glucose intolerance in mice fed a high‐fat high‐sucrose diet

**DOI:** 10.1002/fsn3.1883

**Published:** 2020-09-20

**Authors:** Sangwon Chung, Hyo‐Jin Kim, Hyo‐Kyoung Choi, Jae Ho Park, Jin‐Taek Hwang

**Affiliations:** ^1^ Korea Food Research Institute Wanju‐gun Republic of Korea; ^2^ Department of Food Biotechnology University of Science & Technology Daejeon Republic of Korea

**Keywords:** diosmetin, diosmin, dyslipidemia, fat accumulation, glucose intolerance

## Abstract

This study compared the effects of diosmin and its aglycone, diosmetin, on body weight, liver fat, serum cholesterol, and glucose intolerance in male C57BL/6 mice fed a high‐fat high‐sucrose (HFHS) diet for 12 weeks. The mice were divided into four groups that received the following diets: normal diet (ND), HFHS diet, HFHS diet with 0.5% diosmin, and HFHS diet with 0.5% diosmetin. The body weight increased significantly in the HFHS diet group but decreased significantly in the HFHS diet with 0.5% diosmin group. The diosmin and diosmetin treatment inhibited fat accumulation in liver and epididymal tissues, and improved glucose intolerance by lowering glucose levels during a glucose tolerance test; these effects were greater in the diosmin group than those in the diosmetin group. Furthermore, only diosmin significantly ameliorated dyslipidemia, by reducing TC and LDL‐C levels, while diosmetin had little effect on these parameters. Taken together, the results showed that diosmin and diosmetin can prevent fat accumulation and glucose intolerance; however, diosmin was more effective and also showed an antidyslipidemic effect.

## Graphical abstract

Diosmin and diosmetin improved major parameters of metabolic disorders including fat accumulation and glucose intolerance. However, the effects of diosmin supplement seem to be greater, particularly in terms of the antihyperlipidemic effect.

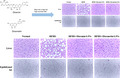

## 1 INTRODUCTION

Diosmin (3′,5,7‐trihydroxy‐4′‐methoxyflavone‐7‐rhamnoglucoside) is a naturally occurring flavonoid glycoside derived from citrus fruits (Benavente‐Garcia & Castillo, 2008; Campanero et al., 2010). Diosmin was first isolated in 1925 from the leaves of *Scrophularia nodosa* L. and is currently produced via dehydrogenation of hesperidin (Feldo et al., 2018). Diosmetin (3′,5,7‐trihydroxy‐4′‐methoxyflavone), a flavone aglycone and the major metabolite of diosmin, can be independently isolated from the leaves of *Olea europaea* L (Meirinhos et al., 2005). When administered, diosmin is converted to diosmetin after hydrolysis by intestinal microflora enzymes, and subsequently absorbed into the circulation (Campanero et al., 2010; Tanrikulu et al., 2013).

Diosmin and diosmetin are known to have several pharmacological effects. According to Ciolino et al, both diosmin and diosmetin have long been known as natural chemopreventive compounds (Ciolino, Wang, & Yeh, 1998). Diosmin is also widely used as an anti‐inflammatory agent in cases of cardiac and hepatic injury. It has been observed that diosmin has vascular–protective functions, improving venous tone, protecting capillary bed microcirculation, reducing capillary permeability, and activating endothelial adhesion molecules and neutrophils (Bergan, Schmid‐Schonbein, & Takase, 2001; Le Devehat, Khodabandehlou, Vimeux, & Kempf, 1997; Manthey, 2000; Ramelet, 2000, 2001). With regard to hepatic damage, diosmin has been shown not only to mitigate ethanol‐induced hepatotoxicity by restoring alanine transaminase (ALT), aspartate transaminase (AST), and lactate dehydrogenase (LDH) levels, and downregulating tumor necrosis factor (TNF)‐α expression and nuclear factor (NF)‐κB activation, but also to minimize methotrexate‐induced hepatic necrosis by suppressing proinflammatory cytokines such as interleukin (IL)‐1β and IL‐6, and tissue levels of malondialdehyde (MDA) and nitric oxide (NO) in several in vivo studies (Abdel‐Daim, Khalifa, Abushouk, Dkhil, & Al‐Quraishy, 2017; Tahir et al., 2013). Diosmetin has also been shown to play an anti‐inflammatory and hepatoprotective role in an in vivo model by suppressing MDA, inducible nitric oxide synthase (iNOS), prostaglandin E2 (PGE_2_), and cyclooxygenase‐2 (COX‐2) expression, NF‐κB signaling pathway activity, hepatocyte apoptosis, and the activities of ALT and AST (Yang et al., 2017).

In addition to the above biological processes, antihyperglycemic activities of diosmin and diosmetin have been observed. In in vivo diabetes models, diosmin improved glucose homeostasis and insulin sensitivity (Gopalakrishnan, Iyyam Pillai, & Subramanian, 2015), and alleviated glucose levels via induction of β‐endorphin secretion (Hsu, Lin, Cheng, & Wu, 2017). Likewise, the seed of the *Artocarpus heterophyllus* fruit, which contains flavonoids including diosmetin, has been shown to possess potential antiglycation activity, which is crucial in the management of diabetes (Shakthi Deve, Sathish Kumar, Kumaresan, & Rapheal, 2014). In particular, diosmin exhibited antihyperlipidemic properties by attenuating the accumulation of lipids such as total cholesterol (TC) and low‐density lipoprotein cholesterol (LDL‐C) through free radical scavenging (Queenthy & John, 2013).

As demonstrated in the above studies, the mechanisms by which diosmin and diosmetin prevent metabolic disorders are similar. However, these natural bioactive compounds have only rarely been studied simultaneously in the same in vivo model. Therefore, this study compares the effects of diosmin and diosmetin on fat accumulation, dyslipidemia, and diabetes in a mouse model of obesity induced by a high‐fat high‐sucrose (HFHS) diet.

## 2 MATERIALS AND METHODS

### 2.1 Animal experiments

All animal experiments were performed at the Korea Food Research Institute (KFRI) according to a protocol approved by the KFRI Institutional Animal Care and Use Committee (Wanju, Republic of Korea; approval number: KFRI‐M‐19025). Five‐week‐old male C57BL/6 mice were obtained from Orient Bio (Gwangju, Republic of Korea) and housed for 1 week for acclimation. The mice were then randomly divided into the following four groups of 10 mice each (3–4 mice/cage): (a) normal diet (ND); (b) HFHS; (c) HFHS diet with 0.5% diosmin; and (d) HFHS diet with 0.5% diosmetin. The diets were purchased from Research Diet, and detailed compositions of each diet are provided in Table 1. Diosmin and diosmetin were purchased from BLDpharm and mixed with the diets. All mice were housed in a climate‐controlled environment with a temperature of 22°C and relative humidity of 50%, under a 12‐hr light/dark cycle with free access to autoclaved tap water for 12 weeks.

**TABLE 1:** Composition and ingredient of the high‐fat high‐sucrose (HFHS) diet

Class	Ingredient	Weight (g)	Caloric value (kcal)
Protein	Casein, lactic, 30 mesh	195	780	
Protein	Methionine, DL	3	12	
Carbohydrate	Sucrose, fine granulated	341	1,364	
Carbohydrate	Lodex 10	100	400	
Carbohydrate	Starch, corn	50	200	
Fiber	Cellulose	50	0	
Fat	Butter, anhydrous	200	1,800	
Fat	Corn oil	10	90	
Mineral	Mineral mix	17.5	0	
Mineral	Calcium phosphate, dibasic	17.5	0	
Mineral	Calcium carbonate, light^a^	4	0	
Vitamin	Choline bitartrate	2	0	
Vitamin	Vitamin mix	10	40	
Antioxidant	Ethoxyquin	0.04	0	
Special	Cholesterol	1.5	0	
Total		1,001.54	4,686	

^a^ United States Pharmacopeia reference standard.

Food was provided ad libitum, and food intake was quantified once a week by calculating the amount left in the food grid. The body weight of the mice was measured at the beginning of the experiment and on the weekly feeding day, alongside the food intake measurement. After 12 weeks, the mice were sacrificed, and blood samples were collected from the heart after ether anesthesia. Liver, epididymal fat, and retroperitoneal fat were weighed and fixed in 4% formalin after laparotomy.

### 2.2 Hematoxylin and eosin (H&E) staining

Liver tissues and epididymal fat were embedded in paraffin after fixation in 4% formalin, and then cut into 5‐μm‐thick sections and stained with H&E. Fat and lipid accumulation in the prepared tissues was assessed using an optical microscope.

### 2.3 Glucose tolerance test (GTT)

A GTT was performed during week 11 of the experiment. The fasting blood glucose levels of the mice were measured using blood obtained from the edge of the tail using an Accu‐Chek Performa blood glucose meter (Roche Diabetes Care, Inc.) after more than 10 hr of starvation. Subsequently, the fasting glucose level was measured and blood glucose levels were measured again at 30, 60, 90, 120, and 180 min after administering a 200 µl/kg body weight glucose solution to each mouse. The area under the curve (AUC) of the glucose levels was also calculated.

### 2.4 Measurement of blood lipid parameters

Whole blood samples were collected from all mice, and the serum was separated. The lipid parameters were analyzed using appropriate kits and analysis methods. TC and high‐density lipoprotein cholesterol (HDL‐C) were analyzed using an ELISA kit (Asan, Seoul, Republic of Korea). LDL‐C was calculated by subtracting HDL‐C from TC.

### 2.5 Statistical analyses

One‐way analysis of variance (ANOVA) followed by Duncan's test was performed using SAS software (ver. 9.4; SAS Institute). All values are presented as the mean ± standard error (SE). Differences were considered statistically significant at *p* < .05.

## 3 RESULTS

### 3.1 Diosmin and diosmetin inhibit weight gain and fat accumulation

**FIGURE 1:**
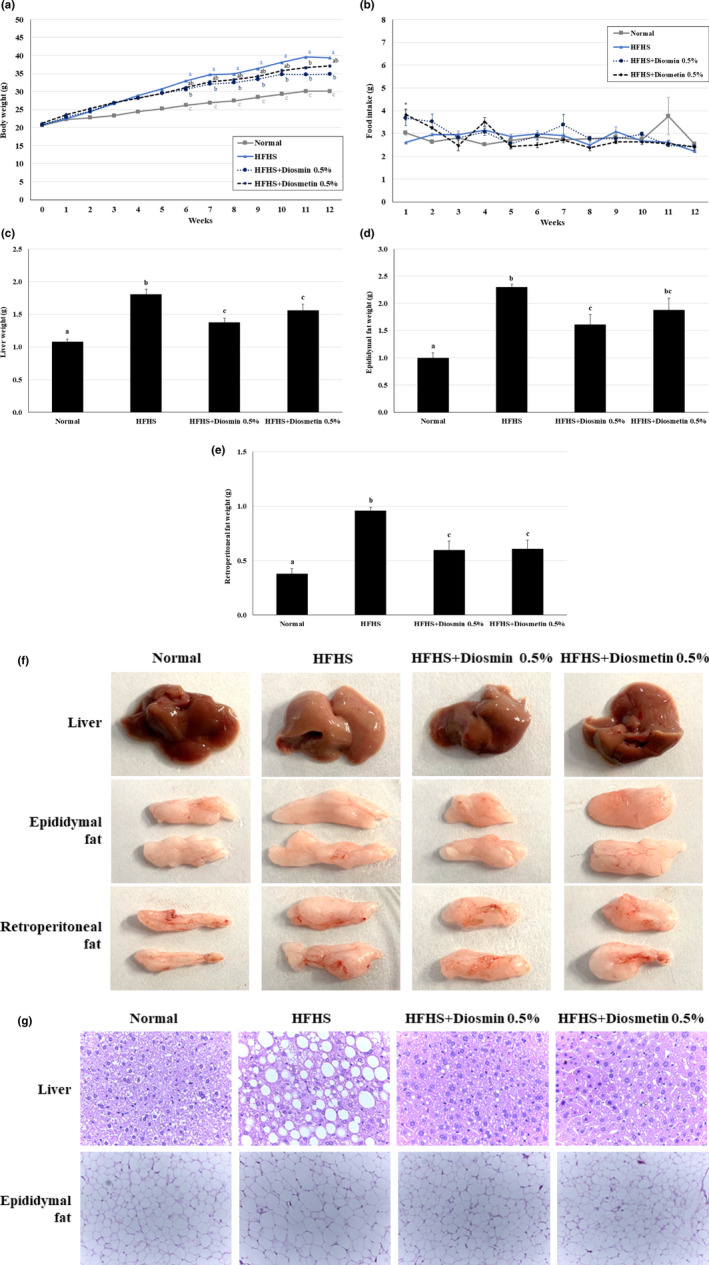
Effects of diosmin and diosmetin on weight gain and fat accumulation in mice fed a HFHS diet. (a) Body weight was higher in mice fed a HFHS diet than in mice fed ND, and supplementation with 0.5% diosmin and 0.5% diosmetin decreased the body weight of HFHS‐fed mice. However, a significant difference between the HFHS diet and HFHS diet with diosmin groups was seen. (b) Food intake was not significantly different between the mice fed a HFHS diet and those on a HFHS diet supplemented with diosmin or diosmetin, except during the first week. (c) Liver weight increased in mice fed a HFHS diet compared with the ND group, but significantly decreased in mice fed a HFHS diet supplemented with diosmin or diosmetin. (d) Epididymal fat weight was significantly higher in HFHS‐treated mice. Diosmin supplementation significantly reduced the epididymal fat accumulation induced by a HFHS diet. Diosmetin treatment did not affect the epididymal fat weight. (e) The retroperitoneal fat weight was significantly higher in HFHS‐treated mice. In addition, both diosmin and diosmetin significantly reduced the retroperitoneal fat content induced by a HFHS diet. (f) Greater fat accumulation in liver, epididymal, and retroperitoneal tissues was observed in HFHS‐fed mice based on visual observation. However, this effect was attenuated by diosmin and diosmetin supplementation. (g) Histological examination of liver and epididymal fat tissues after H&E staining showed that the HFHS diet led to an increase in fat accumulation, which was subsequently reduced by decreasing the size of the lipid droplets in those tissues when the diet was supplemented with diosmin or diosmetin. Values are presented as mean ± SE based on ANOVA (*n* = 10 mice per group). The different lowercase letters indicate significant differences among all groups, and the superscript (*) indicates significant differences between the HFHS group and both the diosmin and diosmetin groups, *p* < .05

We investigated whether supplementation with diosmin or diosmetin can attenuate weight gain in mice fed a HFHS diet. The body weights of mice fed a HFHS diet were significantly higher compared with those of the ND group animals, and those of HFHS diet groups with diosmin and diosmetin were lower than those of the HFHS diet mice; however, a significant difference was only observed between the HFHS and diosmin treatment group at 12th week (Figure 1a). No significant difference in food intake was observed between the HFHS group and either the diosmin or diosmetin treatment groups over the 12 weeks, except for the first week (Figure 1b).

The effects of diosmin and diosmetin on fat accumulation in the liver and epididymal and retroperitoneal fat were also investigated. The liver weight and fat level were increased in mice fed a HFHS diet group compared with the ND diet group, and treatment with diosmin and diosmetin significantly attenuated those effects. The liver weight was lower in the diosmin‐supplemented group compared with the diosmetin‐supplemented group. Furthermore, mice fed a HFHS diet with diosmin or diosmetin had lower levels of epididymal and retroperitoneal fat than mice fed a HFHS diet group. However, supplementation with diosmetin only significantly reduced the retroperitoneal fat and weight while supplementation with diosmin reduced both the epididymal and retroperitoneal fat weight (Figure 1c–f). H&E staining of liver and epididymal tissue was conducted to examine histological alterations; it was observed that diosmin and diosmetin supplementation decreased the size of lipid droplets in the liver and epididymal fat tissue, and attenuated fat accumulation in those fat tissues (Figure 1g). Based on the above results, diosmin and diosmetin supplementation is favorable for inhibiting weight gain and fat accumulation in liver and epididymal tissues; diosmin had a significantly greater effect, especially in the liver.

### 3.2 Diosmin and diosmetin lower lipid parameters

**FIGURE 2:**
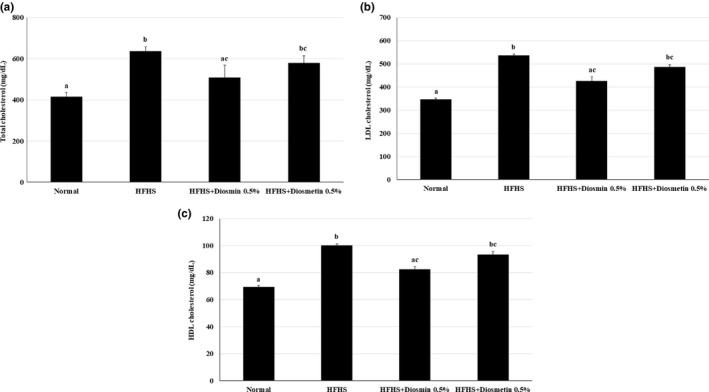
Effects of diosmin and diosmetin on lipid parameters in mice fed a HFHS diet. Diosmin and diosmetin lowered the levels of serum lipids, including TC and LDL‐C, in mice fed a HFHS diet. The levels of TC (a), LDL‐C (b), and HDL‐C (c) were significantly higher in mice fed a HFHS diet than in mice fed the ND, but diosmin consumption significantly reduced TC and LDL‐C levels. Diosmetin consumption slightly reduced the TC and LDL‐C levels. Values are presented as mean ± SE based on ANOVA (*n* = 10 mice per group). The different lowercase letters indicate significant differences among all groups, *p* < .05

Major lipid parameters including the TC, LDL‐C, and HDL‐C levels were analyzed in blood from the sacrificed mice. All lipid parameters were increased in the HFHS‐treated mice compared with the ND mice. The mice fed a HFHS diet supplemented with diosmin showed significantly reduced levels of serum TC and LDL‐C, but those supplemented with diosmetin did not. The serum HDL‐C level did not improve after treatment with either diosmin or diosmetin (Figure 2a–c). Our results suggested that diosmin can lower the cholesterol level increases induced by a HFHS diet.

### 3.3 Diosmin and diosmetin improve glucose intolerance

**FIGURE 3:**
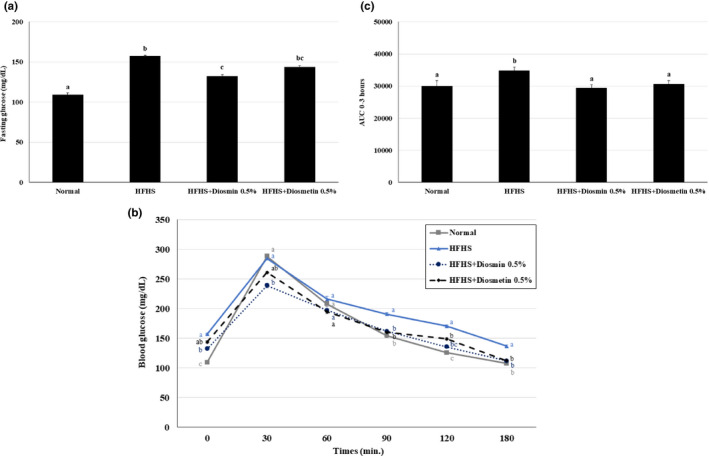
Effects of diosmin and diosmetin on glucose intolerance in mice fed a HFHS diet. (a) The fasting glucose level after more than 10 hr of fasting in mice fed a HFHS diet was higher than that in the ND group. Diosmin treatment, but not diosmetin treatment, significantly decreased the fasting glucose level. The GTT (b) and AUC (c) results over 3 hr showed that diosmin and diosmetin supplementation attenuated the increase in glucose level induced by the HFHS diet. Values are presented as mean ± SE based on ANOVA (*n* = 10 mice per group). The different lowercase letters indicate significant differences among all groups, *p* < .05

To examine the effects of diosmin and diosmetin on glucose metabolism, glucose levels were analyzed and a GTT was conducted after the mice had fasted overnight. The fasting glucose level was higher in the HFHS diet group than in the ND group, and significantly lower in the group fed a HFHS diet supplemented with diosmin. The fasting glucose level in the group fed a HFHS diet supplemented with diosmetin was also lower than that of the HFHS group, but a significant difference was not observed (Figure 3a). The glucose levels during the GTT and AUC of the glucose levels over the 3‐hr GTT indicated that diosmin and diosmetin supplementation significantly mitigated hyperglycemia in mice fed a HFHS (Figure 3b–c). The levels of glucose in the GTT and the AUC of the diosmin treatment group were lower than those of diosmetin treatment group. These results indicate that diosmin and diosmetin attenuate glucose intolerance by lowering the elevated glucose levels induced by a HFHS diet.

## 4 DISCUSSION

We compared the effects of diosmin and diosmetin in several metabolic disorders using an in vivo mouse model of HFHS diet‐induced obesity. Our results show that the body weight was significantly increased in mice fed a HFHS diet, while it was decreased in mice fed a HFHS diet with 0.5% diosmin.

Flavonoids, a type of phenolic compound, are widely known to exert physiological actions in the human body, and are poorly absorbed in the human intestine due to the form of glycosides in the natural state (Murota, Nakamura, & Uehara, 2018). Thus, flavonoids must be hydrolyzed into an aglycone form by enzymes, such as α‐glucosidase and β‐glucosidase, to maximize absorption and physiological efficacy (Rozanska & Regulska‐Ilow, 2018). Flavonoid aglycones penetrate easily through the lumen into the blood due to their high lipophilicity and low molecular weight (Murota et al., 2018). However, flavonoid glycosides exhibit low permeability due to their higher hydrophilicity and larger molecular weight (Hostetler et al., 2012; Murota et al., 2018). Hostetler et al. reported significantly higher absorption for aglycone‐ versus glycoside‐rich diets (Hostetler et al., 2012).

The flavonoid diosmin is hydrolyzed into diosmetin by intestinal microflora and then absorbed; only diosmin has poor solubility and bioavailability (Russo, Chandradhara, & De Tommasi, 2018). Diosmin and diosmetin are structurally different, and it has been reported that they also differ in absorption kinetics (Campanero et al., 2010; Russo et al., 2018). In the present study, mice fed diets supplemented with diosmin and diosmetin showed similar weight gain in the initial week, but diosmin was more effective than diosmetin in reducing body weight in subsequent weeks. These results contrast with previous studies indicating that diosmetin is more effective, in terms of absorption and its physiological actions, due to its aglycone structure (Bredsdorff et al., 2010; Russo et al., 2018); at least in our study, the effects of diosmin did not appear to be related to the rate of absorption. Interestingly, several studies have suggested that glycosides have superior physiological activity to aglycones. It is also reported that hesperidin and neohesperidin exhibited higher inhibitory efficacy on viral infection than their aglycone, hesperetin (Xiao, 2017). Similar to our results, diosmin and rutin have also been reported to inhibit viral infection, while their aglycones, diosmetin and quercetin, respectively, showed no such inhibitory effects (Xiao, 2017). Tewtrakul et al. have also suggested that flavonol glycosides exert a greater inhibitory effect against HIV‐1 RDDP than their aglycones, quercetin, and kaempferol (Tewtrakul, Nakamura, Hattori, Fujiwara, & Supavita, 2002). Several studies have demonstrated that the higher bioactivities of flavonoid glycosides arise from higher plasma levels compared with aglycones, and a longer mean residence time (Xiao, 2017). In the present study, both diosmin and diosmetin inhibited fat accumulation in the liver, epididymal, and retroperitoneal tissues, and improved glucose intolerance by lowering the glucose level during a GTT. However, diosmin had greater preventive effects with respect to fat accumulation in the liver and glucose intolerance than diosmetin. Furthermore, only diosmin significantly alleviated dyslipidemia by reducing TC and LDL‐C levels; diosmetin had little effect on these markers. Therefore, the higher bioactivity of diosmin, a glycoside, compared with diosmetin seems to arise from higher plasma levels and a longer mean residence time.

Further evidence for this result arises from the difference in antioxidant capacity between glycosides and aglycones. Several studies have reported that the antioxidant capacity of flavonoid glycosides is higher than that of flavonoid aglycones. Quercetin, which is the aglycone of many flavonoid glycosides, shows weaker antioxidant activity than the glycosides, quercitrin and isoquercitrin. However, the cellular antioxidant capacity of quercetin is stronger than that of its glycosides. It is assumed that the higher permeability of aglycones, and the particular receptors for galactose in the cell membrane, underlies this difference in cellular antioxidant capacity between quercetin and its glycosides (Choi, Tai, Cuong, Kim, & Jang, 2012). Flavonoid glycosides have also been shown to exert similar or greater levels of anti‐inflammatory activity than their aglycones in in vivo studies (Xiao, 2017). For example, oral kaempferol 3‐O‐glucoside and kaempferol 3‐O‐rutinoside showed high inhibition of neuroinflammation and ischemic brain injury by attenuating signal transducer and activator of transcription 3 (STAT3) and NF‐κB pathway activation in an in vivo study (Yu et al., 2013). Similar or weaker inhibitory effects of several glycosides of kaempferol in murine microglia BV‐2 cells have also been observed compared with their aglycones (Kwon et al., 2004). Taken together, the stronger antioxidant and anti‐inflammatory effects of glycosides compared with aglycones, except at the cellular level where the reverse is true, may be associated with the longer plasma residence times of glycosides. This should be borne in mind when interpreting our study results, where the effects of diosmin and diosmetin were simultaneously examined in plasma levels in vivo, not at the cellular level.

Another study also provided insight into the effects of diosmin, and the difference in its metabolic profile compared with that of diosmetin (which might be an important factor in its physiological activity; Chen et al., 2019). In a rat model, a total of 64 metabolites for diosmin were identified, versus 46 for diosmetin. Interestingly, demethoxylation, decarbonylation, dihydroxylation, and dehydroxylation were found to be pathway‐specific for diosmin (Chen et al., 2019). Other potential mechanisms of action for diosmin and diosmetin should be explored by examining the characteristics of their metabolites. For example, it has been reported that the hydroxyl groups of diosmetin have differential effects on the expression of inflammatory activities depending on the type of cytokine (Zaragoza, Villaescusa, Monserrat, Zaragoza, & Alvarez‐Mon, 2020).

Finally, diosmin and diosmetin had preventive effects on fat accumulation of liver and fat tissues, and in particular, diosmin had antihyperlipidemic effect in this study. However, the antihyperlipidemic effect was only limited to TC and LDL‐C levels because there was no significant difference in other lipid parameters such as triglyceride (TG) levels among mice groups (data not shown). In addition, quantitative analysis of serum lipid levels of liver and fat tissues was not conducted. There are several evidences that serum lipid levels such as TG are associated with liver fat or other fat tissues (Huang et al., 2015; Valkov, Ivanova, Alexiev, Antonov, & Mateva, 2017). Thus, in‐depth experiments with more lipid parameters and analysis for the association of serum lipid levels and fat tissues should be conducted in the further study.

## 5 CONCLUSION

Diosmin and diosmetin improve major parameters of metabolic disorders including fat accumulation and glucose intolerance, but the effects of the former supplement seem to be greater, particularly in terms of the antihyperlipidemic effect. The greater inhibitory effect of diosmin on features of metabolic disorders compared with diosmetin can be explained by its higher bioactivity, which is in turn caused by the higher plasma levels associated with a longer residence time in plasma, despite its poor absorption rate and distinct metabolites. This study provides a compelling rationale for further research comparing the effects of diosmin and diosmetin, and investigating the potential for developing plant‐based antidiabetes and antidyslipidemic agents.

## CONFLICT OF INTEREST

No conflicts of interest exist in relation to this study.

## ETHICAL STATEMENTS

All animal experiments were performed according to a protocol approved by the Korea Food Research Institute Institutional Animal Care and Use Committee (Wanju, Republic of Korea; approval number: KFRI‐M‐19025).
